# PPARs and Anticancer Therapies

**DOI:** 10.1155/2010/536415

**Published:** 2010-06-08

**Authors:** Michael E. C. Robbins, Christine Linard, Dipak Panigrahy

**Affiliations:** ^1^Department of Radiation Oncology, Brain Tumor Center of Excellence, Comprehensive Cancer Center, Wake Forest University School of Medicine, Winston-Salem, NC 27157, USA; ^2^Institute for Radioprotection and Nuclear Safety, 92262 Fontenay aux Roses Cedex, France; ^3^Vascular Biology Program, Children's Hospital, Harvard Medical Center, Boston, MA 02115, USA; ^4^Department of Pediatric Oncology, Dana-Farber Cancer Institute, Harvard Medical School, Boston, MA 02115, USA

Welcome to this special issue of PPAR research, PPARs, and anticancer therapies. Peroxisomal proliferator-activated receptor (PPAR) *α*, *δ*, and *γ* are members of the nuclear hormone receptor superfamily of ligand-activated transcription factors first identified some 20 years ago. Although initial studies focused on their role in regulating cellular metabolism, there has been an increasing appreciation for PPARs' role in regulating a wide variety of biological processes, particularly inflammation and cancer. Modulation of these processes is of particular importance with regard to increasing the therapeutic outcome for cancer patients. This special issue brings together reviews and research articles that focus on the role of PPARs with respect to prognostic outcome, anticancer activity, and normal tissue morbidity, thus demonstrating the exciting therapeutic opportunities that are within our grasp. 

The ability of PPARs to promote or inhibit cancer has been discussed for some time. In this special issue of PPAR research, the first section contains two research articles that focus on the role of PPAR*γ* in malignant melanoma. A mechanistic study described by Klopper and colleagues illustrates how the response of poorly differentiated melanoma cells to PPAR*γ* and retinoid X receptor agonists is mediated through the calcium binding protein S100A2. The necessary presence of S100A2 for the maximum antiproliferative effect of PPARs indicates a potential mechanism of tumor response or resistance to PPAR*γ*-directed anticancer therapy. Additional support for a therapeutic role for PPAR*γ* comes from tissue microarray analyses of normal and tumor tissue from patients with malignant melanoma described by Meyer and colleagues. They report that PPAR*γ* and cyclooxygenase 2 (Cox2) are stage-dependent prognostic markers of malignant melanoma. Moreover, in metastatic malignant melanoma, PPAR*γ* expression may predict for response to bimodulatory stroma-targeted therapy combining Cox/PPAR targeting with metronomic, low-dose chemotherapy. In addition, this article opens the concept of the stroma-tumor interaction complexity.

These research articles are followed by five reviews that demonstrate the potential ability of PPARs to increase the therapeutic window for cancer patients. All of these items strengthen the growing evidence that PPARs' ligands serve as coadjuvants in protection of healthy tissue and in enhancing anticancer therapies. With ongoing improvements in cancer therapy and health care, the population of long-term cancer survivors continues to grow. In the US approximately 62% of adult and over 75% of pediatric cancer patients survive beyond 5 years. For this growing population, late effects of anticancer therapies pose a significant risk and can adversely affect the quality of life of these individuals. Ramanan et al. discuss the growing problem of radiation-induced brain injury, particularly cognitive impairment, observed in patients who survive 6 months or more after partial or whole-brain irradiation (WBI) for primary or metastatic brain cancer. Recent studies suggest that the pathogenesis of radiation-induced brain injury involves WBI-mediated increases in oxidative stress and/or inflammatory responses in the brain. PPAR agonists can cross the blood brain barrier, have well-described anti-inflammatory and neuroprotective properties, and as reported in this review do appear to modulate late radiation-induced brain injury, including cognitive impairment, in rodent models. 

PPAR-mediated modulation of radiation-induced gastrointestinal toxicity is similarly reviewed by Linard and Souidi. Radiation-induced gastrointestinal morbidity for the treatment of pelvic and abdominal tumors is more common than generally recognized and can negatively impact the patient's quality of life. Although the role of PPAR*γ* in colon cancer has been investigated for several years, relatively little is known regarding its potential to protect the gut against radiation-induced injury. This review discusses the effects of abdominal radiation on PPARs, their role and function in radiation-induced toxicity, and the possibility of using PPAR agonists as radioprotectors.

Translating these findings to the clinic is predicated by demonstrating that PPAR agonists will not similarly protect cancer cells. The therapeutic implications of using PPAR agonists in the treatment of human neuroepithelial tumors, including astrocytomas, the highly aggressive glioblastoma multiforme, and pediatric neuroblastomas, are reviewed by Benedetti and colleagues. The majority of malignant neuroepithelial tumors have poor prognosis, and despite multimodality therapy consisting of surgery, radiation therapy, and chemotherapy, long-term survival rates remain poor. These tumor cells express PPARs; thus natural and synthetic PPAR ligands may represent promising adjuvant therapeutic agents when used with existing anticancer therapies. Simpson-Haidaris and colleagues highlight the potential uses for PPAR*γ* agonists in anticancer therapy, with special emphasis on their role as an adjuvant or in combined therapy in the treatment of hematological malignancies found in the vasculature, marrow, and eyes. In addition, they review that the potential roles of PPAR*γ* and its ligands may play in modulating cancer-associated angiogenesis and tumor-stromal microenvironment crosstalk in the bone marrow. Finally, Wagner and colleagues review how PPAR agonists and retinoids can inhibit osteosarcoma proliferation, induce apoptosis, and inhibit tumor growth by promoting osteoblastic terminal differentiation. PPAR agonists have the potential to be used not only as adjuvant therapeutic drugs for osteosarcoma but also as chemopreventive agents for those patients who undergo resection of primary bone tumors in order to prevent local recurrence and/or pulmonary metastasis. 

In conclusion, we hope that you will find these recent advances in the potential role that PPARs and their agonists play in anticancer therapies both informative and exciting. We thank many authors who have contributed to the articles in this special issue and encourage you to further stimulate your research into the role of PPARs and cancer. The millions of individuals who are impacted either directly or indirectly by cancer deserve no less. 

*Michael E. C. Robbins* *Michael E. C. Robbins* *Christine Linard* *Christine Linard* *Dipak Panigrahy* *Dipak Panigrahy*

